# Ligation of Arteries—Farabeuf

**Published:** 1874-08

**Authors:** 


Ligation of Arteries. An operative manual. By Dr. L. H. Farabeuf, Anatomical Assistant to the Faculty, formerly Interne of Hospitals of Paris. Translated by John D. Jackson, M.D., of Danville, Ky. With engravings. Philadelphia: J. B. Lippincott & Co., 1874. 12mo., flexible cloth; pp. 157. Price $1.75.

We know of no more valuable accomplishment for the surgeon than readiness and dexterity in the ligation of arteries. We are quite sure that many precious lives are annually lost for the lack of this accomplishment, which it is the sole object of the little manual to impart. In laying it before the American profession, Dr. Jackson has done an acceptable service. The volume is handsomely gotten up by the publishers, in a form that may readily be slipped into the coat pocket.

